# The Otteroo: A Case Series Exploring Its Potential to Support Physical Therapy Intervention in Infants with or at Risk for Developmental Delay

**DOI:** 10.3390/healthcare9020109

**Published:** 2021-01-21

**Authors:** Isabel Reed, Stacy Menz, Beth A. Smith

**Affiliations:** 1 Infant Neuromotor Control Laboratory, Division of Research on Children, Youth and Families, Children’s Hospital Los Angeles, Los Angeles, CA 90027, USA; ireed@usc.edu; 2 Starfish Therapies, 1541 Old Bayshore Hwy, Burlingame, CA 94010, USA; stacy@starfishtherapies.com; 3 Developmental Neuroscience and Neurogenetics Program, The Saban Research Institute, 4650 Sunset Blvd MS #135, Los Angeles, CA 90027, USA; 4 Department of Pediatrics, University of Southern California, Los Angeles, CA 90033, USA

**Keywords:** aquatic physical therapy, case series, infant, developmental delay, motor development

## Abstract

The objective of this case series was to examine the potential of the Otteroo as a tool to support physical therapy intervention in infants with or at risk for developmental disability. The Otteroo is a float with potential for use in aquatic therapy sessions or as part of a home exercise program. By tracking the amount of use and caregiver perception of the child’s response, we aimed to generate an understanding of the Otteroo’s potential as a family-based adjunct to physical therapy. Four children at risk of developmental delay participated in this study. The Otteroo was provided for four weeks, with recommendations for use. We used an activity log to track usage and collected survey data of caregiver perception of the child’s response. Activity logs showed that use ranged from 3–7 interactions and a total of 40–99.5 min ($\bar{x}$ = 54.88, SD = 29.75). The survey responses varied as to whether caregivers perceived their children enjoyed the experience. Future research should focus on finding effective methods of encouraging Otteroo use if efficacy of an intervention is to be tested. This initial work provides a foundation for future efficacy research with the Otteroo in children with or at risk for developmental delay.

## 1. Introduction

Infants are initially able to move their lower limbs against gravity in a stepping pattern but “lose” the ability after a few months of life. For many years, researchers believed this pattern of motor development was a result of the maturing nervous system [1]. Zelazo and colleagues [2] found that active exercise in newborns prevented the loss of the “walking reflex” in infants with typical development and attributed the maintenance of the reflex to instrumental learning [3]. Alternatively, Thelen and Fisher proposed that the normal disappearance of the reflex was due to asymmetry in weight gained and increased muscle strength during infant development [4], leading to disappearance of the behavior when the legs became too heavy to lift with the current strength level. A study led by Thelen [5] attributed the maintenance of the reflex and increased stepping found by Zelazo and colleagues to an increase in muscle strength from active exercise, overcoming the discrepancy between weight and muscle strength to enable movement. This was further supported by the finding of decreased stepping with the addition of weights to the legs and increased stepping in water, a reduced mass environment [5]. No one has directly tested the relationship between infant leg strength and weight due to difficulties in experimental design [6] but research has demonstrated that early walking skill is affected by body dimensions [7].

Thelen’s theory that the disappearance of the stepping reflex is related to a relative lack of muscle strength is an early example of the Dynamic Systems Theory applied to motor development [6]. According to the theory, movement is not determined by one dominant system but rather by multiple systems interacting in the context of a specific task or situation [8,9,10]. During development, changes in one system affect the stability of a certain behavior, supporting or leading to the emergence of new behaviors [8,10]. In this example, the decrease in stability of the stepping behavior from infant weight gain leads to disappearance of the stepping reflex [8]. Training is one solution for maintenance of the stepping response [3]. Placing infants in water may provide an opportunity for infants to practice movements and control behavior in ways that are not possible in normal-gravity environments. This type of introduced instability is important for physical therapy as a method of encouraging movement exploration and reorganizing movement patterns [8].

Children with developmental delays and disabilities are often recommended to participate in aquatic therapy to supplement clinic, early intervention, or school-based physical therapy or occupational therapy. Researchers have observed positive effects on gross motor skills of children with cerebral palsy after enrollment in an aquatic aerobic exercise program [11]. However, multiple meta-analyses on the effectiveness of aquatic therapy for motor development in children with cerebral palsy have concluded that more research is necessary before conclusions about the therapy’s efficacy can be drawn [12,13]. The need for more research on aquatic interventions for children was supported by a review of 11 studies with children with neuromotor impairments [14]. Thus, here, we consider an aquatic home exercise program as a supplement to general physical therapy rather than as a main strategy of intervention.

**Figure A1:**
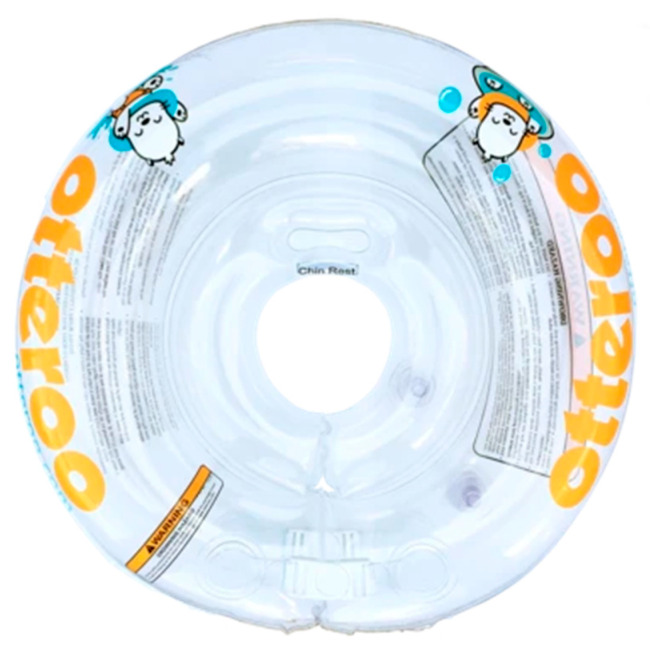
Photograph of the Otteroo LUMI Image from the Otteroo Corporation [19].

This case series explores the Otteroo’s potential as a tool to support physical therapy intervention in infants with or at risk for developmental disability. It has potential to be used in aquatic therapy sessions or as part of a home exercise program. The Otteroo (Figure A1) is a floatie that supports an infant or young child with their head above and their body in water. It allows them to move around in water under direct caregiver supervision and within arm’s reach without the need for the caregiver to physically support the infant or child. It is not intended to be used as a swimming aid or life saving device. As water provides a reduced-gravity environment, it allows infants and young children an opportunity to explore their ability to move and control their bodies more easily than when they are in a ‘typical gravity’ environment. We propose that using the Otteroo to allow movement practice in a reduced-gravity environment will allow infants to perform leg movements they otherwise would not be strong enough to perform and this increased exploration and movement practice may have a positive impact on their development. Before an efficacy trial to compare an Otteroo intervention to a dose-matched alternative intervention can be pursued, we need to understand how families with infants would perceive the device in a physical therapy setting. Accordingly, the goal of this project was to study patterns of Otteroo use with pre-locomotor infants and young children for whom a healthcare provider or caregiver has identified concerns about potential developmental delay. Additionally, we aimed to collect general qualitative information about infant interactions with the Otteroo and measure caregiver perception of infant status over time. Measures of overall developmental status were also taken in order to describe the state of each infant’s development at the time of Otteroo use.

Our goal here was to summarize the theoretical rationale for and explore the potential of the Otteroo as a tool to support physical therapy intervention in infants with or at risk for developmental disability. This initial work provides a foundation for future efficacy research with the Otteroo and similar devices in children with or at risk for developmental delay.

## 2. Materials and Methods

This study received ethical approval from the University of Southern California Health Sciences Institutional Review Board (HS-17-00910). A parent or legal guardian signed an informed consent form prior to their child’s participation. The study was conducted in accordance with the Declaration of Helsinki.

### 2.1. Participants

Participants were recruited June through December of 2018 from Starfish Therapies, a pediatric therapy provider in Burlingame, California. Therapists reached out to caregivers of pre-locomotor children for whom they or a healthcare provider had concerns about potential developmental delay, or children for whom a delay had been identified. Pre-locomotor was defined as children unable to locomote independently more than 4 feet. Potential participants were excluded if they had a diagnosis of Down syndrome or clinical presentation of ligamentous laxity. Those with experience using the Otteroo or without access to an appropriate water source were also excluded. Additionally, infants younger than 8 weeks of age or with body weight of more than 15.88 kg (35 pounds) were excluded due to Otteroo specifications. Children older than 66 months were excluded.

Therapists recruited 4 participants, 2 females and 2 males. At the time of the first visit, participants ranged in age from 99 to 753 days (adjusted for prematurity as appropriate) ($\bar{x}$ = 403, SD = 353). Further information about each participant is provided in Table 1, including weight and age. All participants participated in physical therapy over the course of the study. All therapy and study procedures were overseen by a pediatric physical therapist with 17 years of experience.

#### 2.1.1. Case 1

The first participant was diagnosed with congenital oculomotor apraxia.

#### 2.1.2. Case 2

The second participant was born at 24 weeks of gestation. At birth, he had a germinal matrix bleed and was diagnosed with Stage 2 retinopathy of prematurity. The infant also had an abdominal wall hernia and atopic dermatitis. Additionally, he was diagnosed with patent foramen ovale and chronic lung disease of prematurity. The participant also exhibited gross motor delay, strabismus, and developmental delay. We used adjusted age for this child to account for his preterm birth.

#### 2.1.3. Case 3

This infant was hospitalized for an additional day after birth due to jaundice. She had a preference for right head rotation and left head tilt since birth due to positioning in utero. Additionally, there was a flat spot on her right posterior head.

#### 2.1.4. Case 4

This infant was a twin to Case 3. He had a flat back of his head but was otherwise unremarkable.

### 2.2. Procedure

Research visits took place at Starfish Therapies. Each visit lasted for approximately 30 min. At the first visit, before any study procedures took place, a parent signed an informed consent form for their child’s participation. Descriptive data on the developmental status of each participant were collected at the first and last visit to describe developmental status throughout participation in the study (Alberta Infant Motor Scale and Ages & Stages Questionnaire). The Otteroo was provided to the family at the second visit. Researchers measured the participant’s weight to verify that they were under the specified body weight limit of 15.88 kg (35 pounds) of the Otteroo (LUMI, Otteroo Corp., San Francisco, CA, USA). Additionally, the researcher suggested activities for the Otteroo and ideal durations of use with each of the families. For Case 2, the researcher recommended floating in the pool and using engaging toys. The researcher recommended exploring movement in the water to the caregiver of Cases 3 and 4. Each family was provided with a link to the Otteroo website for more potential activities. At the final visit, after 4 weeks of Otteroo use, activity log data and survey responses were collected.

#### 2.2.1. Primary Measures

The activity log allowed us to measure how much the Otteroo was being used by each child. The activity log collected information on each Otteroo use: date of use, length of interaction, activities during interaction, and additional caregiver comments. Since all interaction with the Otteroo was outside of the researchers’ observation, the activity log provided the only data on the amount of use. Survey questions asked about the participant’s first experience with the Otteroo, changes in behavior or movements, and enjoyment of Otteroo use. The survey also asked about the similarity of the Otteroo to devices previously used in physical therapy, effects of use on family dynamics, and whether the caregiver would recommend the Otteroo to other parents of children with special needs. The qualitative information from the survey and activity log was collected for a better understanding of the quality and makeup of each participant’s interaction with the Otteroo.

#### 2.2.2. Alberta Infant Motor Scale

In order to collect descriptive information about the developmental state of each participant throughout the study, researchers administered the Alberta Infant Motor Scale (AIMS). The AIMS is a standardized, norm-referenced observational scale of motor development [15]. Up to 18 months, the total of scores in specific categories of motor development are referenced to age for a percentile score (Table 2). We chose to use the AIMS (despite having 2 participants above 18 months) as it provided a quantitative description of the motor development of each child and identified which motor skills they are performing.

#### 2.2.3. Ages and Stages Questionnaire

The Ages and Stages Questionnaire (ASQ) was administered in order to describe each child’s overall development to age-based norms across the duration of the study. The ASQ is a standardized, norm-referenced caregiver-report scale of development [16]. Unlike the AIMS, it is not a measure of development over time because the questionnaire changes according to the age of the subject. The ASQ measures development in multiple categories and categorizes the scores based on a cutoff (Table 3).

#### 2.2.4. Therapy

The researchers noted whether the participants were involved in therapy prior to Otteroo use, as well as the frequency and type of therapy. All caregivers reported that their child had the same therapy schedule at the first and second visits.

Participant 1 participated in 5 h of therapy per week. This included 2 h of physical therapy, 1 h of occupational therapy, and 2 h of speech therapy. Participant 2 was involved in therapy for four hours per week. This included 2 h of physical therapy, 1 h of occupational therapy, and 1 h of speech therapy. Participant 3 had 45–60 min of physical therapy and Participant 4 had 60 min of physical therapy.

### 2.3. Safety

Due to the risk of drowning, researchers instructed the caregivers to supervise and be within arm’s reach of the child whenever he or she used the Otteroo in water. Caregivers were also instructed to stop Otteroo use to sooth the participant if they were distressed. If the Otteroo began to deflate, caregivers were instructed to stop activity and re-inflate. While this did not occur, the Otteroo would have been replaced by the researchers if the caregiver reported more than one deflation.

## 3. Results

### 3.1. Activity Log

The total amount of time each infant spent with the Otteroo ranged from 40 to 99.5 min (Table 4). When providing the Otteroo, researchers recommended that Case 1 use the Otteroo for 15–20 min per day, Case 2 use the Otteroo for 15–30 min each day, and Cases 3 and 4 use the Otteroo “as long and as often as possible”. Cases 2, 3, and 4 used the Otteroo about once a week, while Case 1 used the Otteroo four times during the first week, twice during the second, and once during the third. The only participant to refuse use, Case 1 would not interact with the Otteroo during 4 of the caregiver’s 7 attempts. Case 2 only interacted with the Otteroo in the pool, while Cases 1, 3, and 4 interacted with the Otteroo in the bath and pool. For Cases 3 and 4, the first 2 Otteroo interactions were in the bath and the third was in the pool. Case 1 had 4 interactions with the Otteroo in the bath and 1 in the pool with the location of 2 interactions unspecified.

In the additional comments section, each caregiver expanded upon their child’s experience with the Otteroo. The caregiver for Case 1 noted twice that she did not like the Otteroo around her neck. For Case 2, the caregiver described their child’s discomfort with the Otteroo after initially putting it on but enjoyment after relaxing and going into the water. The caregiver for Cases 3 and 4 shared that the infants seemed happy while using the Otteroo, with Case 4 moving around more than Case 3 during their use in the pool.

### 3.2. Survey

The survey responses provide information on each caregiver’s interpretation of their child’s interaction with the Otteroo and their perception of its potential effects. It is important to note here that Cases 3 and 4 have the same caregiver.

The caregivers were asked to compare their child’s first experience with the Otteroo to their experience after a couple of uses. Case 2′s caregiver stated that, the “first time felt restrictive for him but after he went in the water he felt more comfortable”. The caregiver for Cases 3 and 4 noted that Case 4′s movement increased from his first to third interaction with the Otteroo. For Case 1, her caregiver described a persistent struggle to encourage use.

One survey question asked the caregivers “how [the Otteroo] is different from other devices or activities” used in their child’s physical therapy. Cases 1 and 2 had never used comparable devices in therapy. The caregiver for Case 4 noted the fun the infant was able to have with the Otteroo. For Case 3, they described appreciating the differences to previous devices in that the Otteroo can be used in the bath. However, they noted the difficulty of attempting to bathe two infants at once while using the Otteroo.

The survey also asked about each caregiver’s interpretation of their child’s interaction with the Otteroo and if the use affected their family dynamic. Case 1′s caregiver stated that the infant did not like the Otteroo. The caregiver for Case 2 stated that he “enjoyed being in the water since using it” and “likes the anticipation of going in the water”. For Case 3, her caregiver described the participant tolerating the Otteroo without seeming “to love it or hate it”. Case 4′s caregiver stated that they “think he enjoys how kicking allows him to move in the water”. The caregiver for Cases 3 and 4 described the Otteroo creating a “giant fun experience” for their family in the pool.

The final question asked whether the caregivers would recommend the Otteroo to parents of children with special needs. Case 1′s caregiver “recommends for children a lot younger than 1.5 or 2 years old”. The caregiver for Case 2 “highly recommend[s] device to increase your child’s confidence in the water”. Case 3 and 4′s caregiver recommends for general pool use but not necessarily for therapy because the “results [are] still not proven to” her.

## 4. Discussion

The results of this case series suggest that the Otteroo has potential for use in aquatic therapy sessions, as part of a home exercise program, or as an adjunct in addition to therapy services. Survey results indicated an overall positive impression of the Otteroo for the caregivers of Cases 2, 3, and 4. The caregivers recommended introducing it early in life and allowing children to become comfortable with the Otteroo over time. However, the variance in device use, participant reactions, and parent satisfaction demonstrates that future studies on the efficacy of devices like the Otteroo must address several factors.

In our study, families interacted with the Otteroo over 4 weeks and their total use varied from 40 to 99.5 min. Future research should not only have a longer intervention period but should also encourage a greater frequency of use than that observed here. Previous research found that aquatic therapy was beneficial after 36 and 40 weeks of intervention [17,18]. This is a much longer period of time than the 4 weeks of intervention in our current study. However, this study was intended to gather thorough feedback based on multiple interactions with the Otteroo. The duration of intervention was appropriate for that purpose; longer lengths of intervention are required for results beyond the scope of the current study.

To move forward with studies of the Otteroo or similar devices, it will be important to help families introduce the device and scale up its use to a level consistent with providing sufficient practice to support skill development. In regard to frequency, although daily use was encouraged in this study, daily use may present a significant burden for each family, and a schedule of two or three times per week may be more appropriate, especially to start. One way to encourage a greater frequency of use would be to designate a specific time and place for scheduled interactions with the Otteroo. Further, providing a location for Otteroo interaction would expand the population of potential participants to infants with families without bathtubs or access to pools. In this case series, families were encouraged to use the Otteroo in the bath or pool as they saw fit and were able. It would be helpful for future work to investigate the potential importance of consistency of location and potential differences in Otteroo use in different locations.

Additional suggestions for increasing Otteroo use would be to provide a tailored list of activities for each infant, based on development, therapy goals, and/or medical diagnosis. Especially since a majority of caregivers saw the benefits of the Otteroo as a pool toy rather than a therapy device, this may encourage the perception of and interaction with the Otteroo as a tool for physical therapy. All participants in this study participated in more therapy than they did “Otteroo time”. No matter the methodology of future research, an essential component must be extending the study to lengths previously found to have significant effects and encouraging more interaction and use of the device.

The refusal of Case 1 to interact with the Otteroo multiple times is an important consideration for future work. Researchers may need to intervene in the case of multiple negative interactions and provide additional training or recommendations to the caregivers according to the purpose of their work. A better understanding of the interaction of infants with the Otteroo beyond parent report would be beneficial to any future work with the Otteroo. For example, coding videos of multiple Otteroo sessions would provide valuable information on infant movement and behavior during the interaction. This would allow researchers to provide more informed trainings and recommendations as well as design study protocols with a stronger foundation of knowledge.

A limitation of our study was the age and number of participants. Two of the infants were older than the Albert Infant Motor Scale maximum age of eighteen months. Notably, the caregiver for Case 1 attributed the infant’s negative interactions with the Otteroo to her age. As this was a case series, a subject pool with varied age and development was appropriate in order to gather initial data about family experiences and perceptions. The inclusion of diverse infants with and at risk for developmental disability fits the aim of this study to explore how infants with different therapy needs interact with the Otteroo. Future research should set more strict recruitment parameters for the age of the participants based on the measures of development and the age limitations of the Otteroo. Any experimental studies should recruit a sufficiently large number of participants and consider any previous experiences (positive or negative) with swimming or aquatic therapy. One method may be to create a comparison between dose-matched physical therapy and aquatic physical therapy groups. Researchers may also want to narrow recruitment to specific at-risk populations, as aquatic therapy may be more beneficial for infants with specific diagnoses. Future studies should also consider that the Otteroo may be more beneficial during a specific period of development, such as just before the onset of crawling and walking.

## 5. Conclusions

As the first examination of the Otteroo as a tool in physical therapy, this study provides valuable information for future studies of the Otteroo and other physical therapy tools. Our research demonstrates that while the Otteroo has potential as an adjunct to physical therapy, one of the biggest challenges to Otteroo use as a physical therapy tool is identifying and encouraging an appropriate amount of time each family can dedicate to its use. This would be especially important in efficacy studies designed to address the relationship of Otteroo use to motor and overall development. Finally, as with other studies of aquatic physical therapy, future research should address whether increased movement in water translates to improvement in normal-gravity environments.

## Figures and Tables

**Table 1:** Demographic Information at Visit 1 (Week 0).

Case	Sex	Age (Days)	Adjusted Age * (Days)	Weight (kg)
1	Female	662	-	13.2
2	Male	864	753	9.98
3 ^†^	Female	99	-	5.44
4 ^†^	Male	99	-	5.44

* Adjusted age is the age of the child adjusted for preterm birth. ^†^ Twins.

**Table 2:** Alberta Infant Motor Scale (AIMS) Percentile Ranks.

Case	Week	Percentile
1	0	5
	4	5
2	0	5
	4	5
3	0	10
	4	25
4	0	25
	4	50

**Table 3:** Ages and Stages Questionnaire (ASQ) State Relative to Cut Off.

Case	Week	Communication	Gross Motor	Fine Motor	Problem Solving	Personal-Social
1	0	Close	Below	Below	Above	Below
	4	Close	Below	Below	Above	Below
2	0	Below	Below	Below	Below	Below
	4	Below	Below	Below	Below	Below
3	0	Close	Above	Below	Close	Above
	4	Above	Below	Below	Close	Above
4	0	Above	Above	Below	Above	Above
	4	Above	Above	Above	Above	Above

**Table 4:** Interactions with Otteroo from Activity Log.

	Uses	Average Length ^a^	Total Length ^a^
Case 1	7	5	40
Case 2	3	33.17	99.5
Case 3	3	13.33	40
Case 4	3	13.33	40
$\bar{x}$	4	13.72	54.88
SD	2	11.67	29.75

^a^ Values in minutes; $\bar{x}$ = mean; SD = standard deviation.

## Data Availability

Data are contained within this report.
